# Editorial: Microbial influences on aging: insights from the gut microbiome

**DOI:** 10.3389/fragi.2026.1890954

**Published:** 2026-06-17

**Authors:** Christina Tsigalou

**Affiliations:** Faculty of Medicine, School of Health Sciences, Democritus University of Thrace, Alexandroupolis, Greece

**Keywords:** aging, biomarker, diet, gut microbiome, longevity

Ιs aging an inevitable process of biological decline, or can some of its trajectory be shaped by the microbial communities that inhabit the human gut? Could the gut microbiome serve as a biomarker of healthy aging? Through which mechanisms do intestinal microorganisms influence inflammation, metabolism, barrier integrity, and resilience later in life? And to what extent might nutrition and other modifiable lifestyle factors reshape these interactions? The articles gathered in this Research Topic address these timely questions and collectively highlight the gut microbiome as an important contributor to healthy aging and healthspan. As populations worldwide continue to age, understanding how microbial ecology interacts with host physiology has become increasingly relevant for prevention strategies and future therapeutics.

The first study, by Miller et al., expands the discussion beyond the intestine and explores the relationship between microbial imbalance and respiratory vulnerability in older adults. The detection of *Enterobacterales* in the oropharynx of residents in long-term care facilities emerged as a meaningful marker of increased infection risk. Importantly, the authors identified overlap between gut-associated strains and microorganisms colonizing the upper respiratory tract, suggesting possible microbial translocation or shared ecological disruption. These findings emphasize that age-related microbial dysregulation may extend beyond the gut and reflect broader weakening of host defense systems.

Shifting toward prevention and modifiable risk factors, the study by Jones et al. examines how diet may influence intestinal barrier integrity in healthy older adults. Using lipopolysaccharide-binding protein (LBP) as a marker of gut permeability, the authors demonstrate that nutritional quality is closely linked to host–microbiome interactions. Higher fiber intake was associated with a more favorable barrier profile, whereas higher fat intake correlated with less favorable outcomes. This work reinforces the concept that diet is not merely a source of energy, but a key regulator of microbial metabolism, inflammation, and intestinal resilience during aging.

The population-specific and metagenomic dimensions of healthy aging are explored in the study by Almatrafi et al., conducted among healthy Saudi adults. Notably, aging was not portrayed solely as a process of decline, but also as one of adaptation and ecological remodeling. Increased microbial diversity across age groups, together with enrichment of taxa potentially linked to short-chain fatty acid production, suggests that some microbial configurations may support metabolic stability and healthy aging. These findings are especially valuable because they broaden current knowledge beyond Western cohorts and underline the importance of geography, lifestyle, and diet in shaping aging-associated microbiome signatures.

Complementing these observations, Xu et al. investigate mechanistic links between the gut microbiome, bile acid metabolism, intestinal barrier dysfunction, and chronic low-grade inflammation. Their findings show that aging is associated with marked shifts in bile acid profiles alongside microbial changes that may favor pro-inflammatory states. Associations with increased lipopolysaccharide, IL-6, TNF-α, and other aging-related markers support the concept that the gut microbiome–bile acid axis may play a central role in inflammaging. This work helps explain how age-related microbial alterations may translate into systemic physiological consequences and identifies bile acid metabolism as a promising therapeutic target.

Τaken together, the articles in this Research Topic converge on a clear message: aging is not only a chronological process, but also an ecological and metabolic transition shaped in part by the microbiome. From infection susceptibility and nutritional modulation to metagenomic signatures and host–microbial metabolic pathways, these studies suggest that supporting the microbiome may contribute to healthier ageing trajectories and improved healthspan. The insights presented here support the development of innovative strategies—from targeted nutrition and probiotics to personalized microbiome-based interventions—aimed at promoting healthier, more functional, and higher-quality years of life.

